# HIV-1 Viral Load Assays for Resource-Limited Settings: Clades Matter

**DOI:** 10.1371/journal.pmed.0030538

**Published:** 2006-12-26

**Authors:** Wolfgang Preiser, Jan Felix Drexler, Christian Drosten

The paper by Fiscus et al. [1] gives an excellent overview of current technologies to measure HIV-1 viral load (VL), and highlights the problems with finding assays that, whilst affordable and practicable, provide reliable and high-quality results to guide patient management in resource-constrained settings.

One concern, which in our mind does not receive enough attention in the paper, is HIV variability. The assays' ability to reliably quantitate non-B HIV-1 subtypes and strains is of paramount importance, given the predominance of non-B HIV infections in developing countries [2]. Unfortunately, genotype bias has not been assessed systematically for most of the assays described in the paper.

In our recent paper, “Ultrasensitive Monitoring of HIV-1 Viral Load by a Low-Cost Real-Time Reverse Transcription-PCR Assay with Internal Control for the 5' Long Terminal Repeat Domain” [3] (which is found by Fiscus et al.'s PubMed search strategy for “HIV-1 viral load AND resource-limited settings” but presumably just missed the deadline for [1]), we describe an inexpensive assay targeting the conserved long terminal repeat (LTR) domain of HIV-1.

In addition to comparing the assay with three commercial tests using patient samples from Brazil, South Africa, India, and Germany, we also extensively determined its ability to accurately quantitate different HIV-1 subtypes. For this we used two HIV-1 subtype reference panels (National Institute for Biological Standards and Control: HIV-1 M subtypes A, B, C, D, circulating recombinant form [CRF] AE, F, G, H, and HIV-1 N and O; German National Reference Centre for Retroviruses: HIV-1 M subtypes A, B, C, D [2 samples], CRF AE, F, G, and HIV-1 O [2 samples]), as well as 12 plasma samples from patients infected with non-B subtypes, including genotypes not present in the reference panels, e.g., CRFs AE and AG and subtype J, as determined by sequence analysis.

We demonstrated that LTR is highly suitable for the quantification of “exotic” HIV-1 genotypes: For all genotypes, except for groups N and O, the overall results of virus quantification using different assays were highly concordant. For HIV-1 M clades and CRFs, the differences between our assay and the Roche Monitor assay were below 0.5 log; this excellent comparability between the assays is clinically acceptable. The reduced risk of genotype bias makes monitoring of HIV-1 VL feasible, to improve individual patient management as well as hopefully delay the emergence and spread of antiretroviral drug resistance.

Our test is at least as good as commercial tests with regard to performance and technical features: The internal control allows recognition of inhibition and thus falsely low or negative results; its accuracy at least equals that of commercial assays; its analytical sensitivity (down to 32 copies/ml according to industry-agreed evaluation procedures including probit analysis) and broad quantification range are sufficient for both treated and untreated patients; and it is much cheaper than comparable commercial assays—less than US$10 for reagents per sample and below US$20, including the service licence payable to Roche. Its only disadvantage is that it still requires relatively expensive equipment, a certain infrastructure, and skilled personnel. Large-scale feasibility studies have commenced in Brazil, and Stellenbosch University in South Africa is going to set up the assay for viral load testing.

Given the large and sadly still rapidly increasing populations of HIV-1-infected individuals in many resource-constrained countries, together with the drive to provide universal access to antiretroviral treatment and the looming danger of antiretroviral drug resistance as a significant public health problem jeopardising the success of antiretroviral treatment programmes [4,5], the potential benefit of affordable HIV-1 VL assays is enormous. The challenge lies in ensuring that these assays are adequate for the respective situation, particularly with regard to HIV-1 strain diversity.
